# Permanent neonatal diabetes mellitus in China

**DOI:** 10.1186/1471-2431-14-188

**Published:** 2014-07-23

**Authors:** Ke Huang, Li Liang, Jun-feng Fu, Guan-pin Dong

**Affiliations:** 1 Department of Endocrinology, Children’s Hospital of Zhejiang University School of Medicine, 57 Zhugan Xiang, Hangzhou 310003, China; 2 Department of Pediatrics, The First Affiliated Hospital of Zhejiang University, 79 Qingchun Road, Hangzhou 310003, China

**Keywords:** Permanent neonatal diabetes mellitus, Genetic analysis, Therapy

## Abstract

**Background:**

Permanent neonatal diabetes mellitus (PNDM) is a rare disease, which is defined as the onset of diabetes before the age of 6 months with persistence through life. Infants with *KCNJ11* or *ABCC8* genetic mutations may respond to oral sulfonylurea therapy. Currently, there are limited studies about the genetic analysis and long-term follow-up of PNDM.

**Case presentation:**

We report four cases of PNDM. None of the infants or their parents had *INS*, *KCNJ11,* or *ABCC8* genetic mutations. One infant underwent continuous subcutaneous insulin infusion (CSII) and the other infants underwent multiple injections of insulin (MII). In these infants, PNDM persisted from 35 months to 60 months of follow-up. Three infants maintained fairly stable blood sugar levels, and one infant had poor sugar control.

**Conclusions:**

We suggest that all of the infants with PNDM should undergo genetic evaluation. For infants without *KCNJ11* and *ABCC8* genetic mutations, oral sulfonylurea should not be considered as treatment. CSII is a useful method for overcoming the difficulties of diabetes, and it may also improve the quality of life of both infants and their parents.

## Background

Permanent neonatal diabetes mellitus (PNDM), which refers to the onset of diabetes before the age of 6 months with persistence through life, is a rare disorder with an incidence ranging from 1:210,000 to 1:260,000 [1,2]. Together, activating mutations in *KCNJ11* and *ABCC8* genes, that encode the Kir6.2 andsulfonylurea receptor 1 (SUR1) subunits, respectively, account for more than 40% of PNDM cases [3]. Infants with these genetic mutations may benefit from switching to oral sulfonylurea therapy. To date, only few data have been published regarding PNDM in China [4]. Moreover, there were limited studies about the genetic analysis and long term follow-up of PNDM. Herein, we report four cases of PNDM and review the relevant literature.

## Case presentation

### Case 1

**Figure 1:**
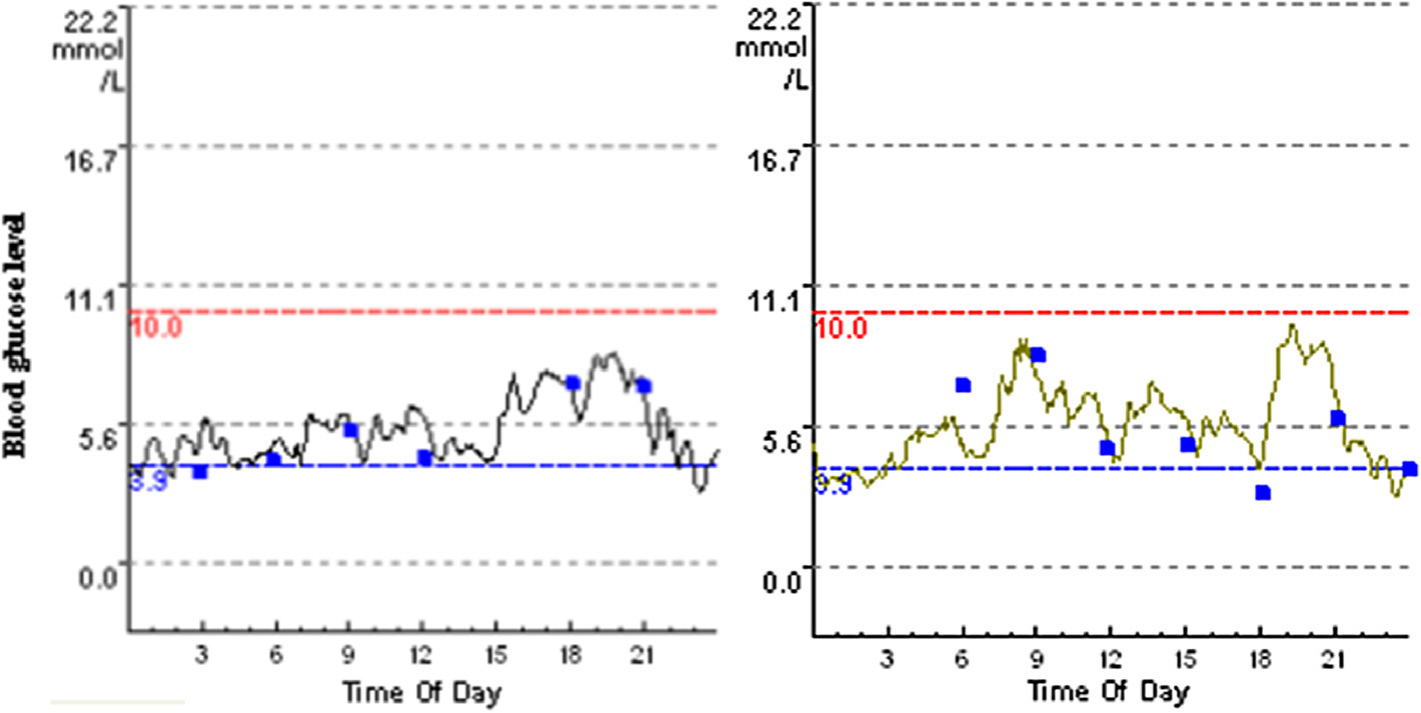
Continuous glucose monitoring system data of the patient treated with CSII (Case 1).

A 14-day-old infant girl was admitted to our hospital with a two-day history of fever and dyspnea. She was conceived by *in vitro* fertilization and embryo transfer from non-consanguineous parents, and was delivered at term with weight appropriate for her gestational age, with no significant perinatal problems. There was no family history of diabetes mellitus or neurological disorders. At presentation, she was in severe respiratory distress with hyperglycemia and metabolic acidosis, her peripheral blood sugar was 39.0 mmol/L. She was subsequently diagnosed with PNDM. Her first arterial blood gas analysis revealed the following parameters: pH 7.114 (normal range: 7.35–7.45), CO_2_ 17.1 mmHg (normal range: 35–48 mmHg), and bicarbonate 5.2 mmol/L (normal range: 21–28 mmol/L). Urinalysis demonstrated 4+ sugars and 4+ ketones. Her glycosylated hemoglobin (HbA1c) and C-peptide levels were 5.9% (normal range: 4.5%–6.3%) and 0.31 ng/mL (normal range: 0.6–3.8 ng/mL), respectively. Serum insulin was 3.1 mIU/L (normal range: 1.9–23 mIU/L). Further investigations showed negative pancreatic autoantibody, and a normal pancreas was identified by ultrasonography. Her temperature normalized progressively on the 3^rd^ day of hospitalization after receiving anti-infection therapy. Upon hospitalization, the infant received insulin therapy. At first, she underwent volume resuscitation and intravenous insulin infusion, then she was administered multiple injections of insulin (MII). She was fed 8 to 12 times a day and the dose of insulin was adjusted based on glucose levels, which fluctuated between 0.9–1.1 U/kg/d. On the 7^th^ hospitalization day, she was given continuous subcutaneous insulin infusion (CSII, Medtronic 712, Minneapolis, MN, USA) treatment and the insulin dose was adjusted according to blood glucose levels yielded by a continuous glucose monitoring system (CGMS gold, Medtronic, Minneapolis, MN, USA) (Figure 1). When she was discharged, the dosages of basal and bolus insulin were 0.6 U/kg/d and 0.2 U/kg/d, respectively, and her blood glucose level stabilized at 4.6–9.1 mmol/L. She is currently 35 months old and remains insulin-dependent. The most recent HbA1c was 6.7%, and she has episodes of hypoglycemia rarely. However, she has a slight development delay. She was 85 cm in height (below the 3^rd^ percentile of the normal length for Chinese girls) and 12.5 kg in weight (in the 15^th^ percentile of the normal weight for Chinese girls) at 35 months of age.

### Case 2

A 37-day-old infant boy was presented to a peripheral hospital with a two-day history of fever and diarrhea. He was the third baby of non-consanguineous parents, and was born without perinatal complication. There was no family history of DM or neurological disorders. At presentation, he was also severely dehydrated, with hyperglycemia and metabolic acidosis. Peripheral blood sugar was 48.5 mmol/L. The first arterial blood gas analysis showed metabolic acidosis. His HbA1c and C-peptide levels were 8.3% and 0.20 ng/mL, respectively. His body temperature normalized and the diarrhea ceased on the second day of hospitalization after receiving anti-infection therapy. Insulin infusion was started immediately and then switched to MII on the 3^rd^ day. An attempt was made to switch to oral glibenclamide therapy using the inpatient protocol described by Pearson *et al.*[5] on the 5^th^ day before the genetic analysis results were obtained. Unfortunately, the infant could not be weaned off insulin. He was subsequently transferred to our center owing to the difficult control of his blood glucose level by using different insulin regimens. He was fed 8 times a day and the dose of insulin was adjusted based on glucose levels, which fluctuated about 1.1 U/kg/d. He was discharged on the 10^th^ hospitalization day when his blood glucose levels stabilized between 8.0–11.4 mmol/L. At 45 months of age, he weighed 14.0 kg. He received MII at 0.71 U/kg/ day, and his most recent HbA1c level was 7.1%, with a few episodes of hypoglycemia.

### Case 3

The clinical features are summarized in Table 1.

**Table 1:** Clinical features of the four infants with PNDM

	**Case 1**	**Case 2**	**Case 3**	**Case 4**
At diagnosis				
Age	14 days	37 days	2 months	38 days
Gender	F	M	M	M
Family history of diabetes	negative	negative	negative	negative
Birth weight	AGA	AGA	AGA	AGA
Blood glucose level (mmol/L)	39.0	48.5	27.0	40.0
HbA1c (%)	5.9	8.3	11.9	8.5
C-peptide (ng/mL)	0.31	0.20	0.25	0.57
Insulin level (mIU/L)	3.1	1.5	0.9	No data
DKA	yes	yes	no	yes
ABG				
pH	7.114	7.250	7.330	6.911
Bicarbonate (mmol/L)	5.2	16.9	25.6	4.5
Pancreatic autoantibody	negative	negative	negative	negative
Urine				
Urine sugar	4+	4+	3+	4+
Urine ketone	4+	4+	+/-	3+
Pancreas ultrasonography	normal	normal	normal	normal
Recent follow-up				
Age	35 months	45 months	54 months	60 months
Insulin injections	CSII	MII	MII	MII
Insulin dosage	Humulin: basal 0.6 U/kg/dbolus 0.2 U/kg/d	Humulin: 0.7 U/kg/ day	Novolin 30R: 0.4 U/kg/ day	Humulin: 0.5 U/kg/day
Hypoglycemia/hyperglycemia	rare	few	sometimes	always
HbA1c (%)	6.7	7.1	8.0	10.5
Combine with other problems	Development delay	No	epilepsy	No

PNDM, permanent neonatal diabetes mellitus; AGA, appropriate for gestational age; HbA1c, glycosylated hemoglobin; ABG, arterial blood gas; CSII, continuous subcutaneous insulin infusion; MII, multiple insulin injections; DKA, diabetic ketoacidosis.

### Case 4

The clinical features are summarized in Table 1.

### Genetic analysis

After obtaining informed consent from parents, genomic DNA from peripheral blood leukocytes was extracted from the infants and their parents using standard methods when the infants were 2 months, 17 months, 25 months, and 4 years of age, respectively. The coding regions and intron/exon boundaries of the *ABCC8*, *KCNJ11,* and *INS* genes were amplified by polymerase chain reaction (primers available on request). Single-strand sequencing was carried out using standard methods on an ABI3730 capillary sequencer (Applied Biosys-tems, Warrington, UK) and sequences were compared with published sequences (NM_000352.2, NM_000525, and NM000207.2) using Clustal X2.0 software (SFI, Dublin, Ireland). The results indicated that only the infant in Case 3 presented a heterozygous mutation in a non-coding area of the *INS* gene. None of the infants or their parents presented any mutations of *KCNJ11* and *ABCC8* genes. This is the reason why the infant in Case 2 was unresponsive to treatment with sulfonylureas. Regretfully, we did not test for other genetic mutations that may also cause PNDM, such as *GCK, IPF1, FOXP3, PTF1A,* and *EIF2AK3*[2].

## Conclusions

NDM is a rare disease, and is classified as TNDM or PNDM if the symptoms are resolved or continuous after 18 months of birth. It was previously regarded as early onset type diabetes; however, studies of its pathogenesis have proved it to be monogenic in origin. In China, the first case of NDM was reported in 1986. In recent years, doctors have paid close attention to the pathogenesis, classification, and new treatments of PNDM.

To the best of our knowledge, *KCNJ11* (~30%), *ABCC8* (~19%), and *INS* (~20%) genes are responsible for the majority of PNDM cases [2]. Sulfonylureas, which bind to SUR1 and close the KATP channel, triggering insulin secretion, were effective and safe in most patients with mutations in *KCNJ11* or *ABCC8* genes [6]. Glibenclamide was chosen for one of the infants based on the inpatient transfer protocol of Pearson et al. [5] before doctors got the genetic analysis results. Unfortunately, the switch from insulin injection to oral sulfonylureas was not successfully achieved. We analyzed the commonest genetic mutations and found no remarkable mutations among these four infants and their parents. However, it is possible that these were triggered by other genetic mutations that we did not analyze. Syndromic forms of neonatal diabetes can be caused by mutations in *FOXP3*, *PTF1A*, *GLIS3*, *NEUROD1*, *RFX6*, *NEUROG3*, *EIF2AK3*, *GATA6*, *SLC19A2*, *HNF1B*, *PAX6*, and *WFS1*. The infants in these cases did not have any syndromic features, and thus, we did not test for those genes. The genetic characteristics of the infant in Case 2 most likely explains why the attempted sulfonylurea treatment failed. Apart from those with *KCNJ11* or *ABCC8* mutations, insulin is the mainstay of treatment of PNDM, although there is evidence that sulfonylurea treatment could worsen beta-cell apoptosis [7]. Therefore, we think that infants with PNDM should be treated with oral sulfonylureas only after the genetic analysis shows *KCNJ11* or *ABCC8* mutations. We suggest that all the patients with PNDM should undergo genetic testing, not only because it can lead to sulfonylureas treatment switching for patients with *KCNJ11* or *ABCC8* mutations, but also because it is useful for genetic counseling.

Furthermore, timely diagnosis and adequate treatments are crucial for PNDM. First of all, the diagnosis of NDM is based on clinical symptoms and lab tests, such as blood glucose and arterial blood gas levels. However, measurement of HbA1c is not suitable for diagnosing diabetes mellitus in infants younger than six months because of the higher proportion of fetal hemoglobin compared with hemoglobin A. Insulin therapy may allow survival and normal development; however, the parents’ ability of subcutaneous insulin injection and skilled home nursing are necessary. It has been reported in China that some infants have died because of poor parental understanding of the importance of insulin treatment and the lack of home nursing care [8]. Infants need extremely small doses of insulin and have a higher risk of hypoglycemia or hyperglycemia, which needs to be avoided because of the high risk of brain damage. The CSII has been used in pediatric diabetes care since 1979. The use of CSII is now increasing among NDM infants [9]. The use of CSII decreases HbA1c values and episodes of severe hypoglycemia, consequently improving the quality of life [10]. CSII also has the significant advantage of delivering very small doses of both bolus and basal accurately. Especially for those newborn infants with diabetes, CSII overcomes the difficulties of diabetes. Some pediatric centers in France have used CSII for the treatment of NDM when the need for insulin has lasted for more than 15 days [11]. Some studies conclude that the therapeutic application of CSII during the neonatal period is safe, effective, more accurate, and easier to manage than injections [10]. Among our four infants, the infant in Case 1 had better treatment compliance, more stable blood glucose levels and better quality of life than the other infants. Unfortunately, CSII can be costly in China and in Cases 2–4 the infants’ families did not have the economic capability to pay for CSII.

In summary, we reported 4 cases of PNDM in which the patients were negative for mutations in *KCNJ11*, *ABCC8,* and *INS* genes. We recommend that all the infants with PNDM should undergo genetic analysis before oral sulfonylurea treatment. Stable blood sugar levels are the key to survival and normal development of these infants. Parents have the skills to be able to administer subcutaneous insulin injections. We consider that CSII is a useful method to overcome the difficulties of diabetes, and it improves the quality of life of both infants and their parents.

### Consent

Written informed consent was obtained from all parents of the infants for publication of this Case report and any accompanying images. A copy of the written consent is available for review by the Editor of this journal.

### Ethics

Written informed consent was obtained from the patients’ parents for their blood collection and genetic analysis. Genetic analyses were also approved by the ethics committee at our hospital.

## Competing interests

The authors declare no conflict of interest.

## Authors’ contributions

KH: acquisition, analysis and interpretation of the patient data and drafting of the manuscript. LL: conception and design this study. JF: revised the manuscript critically and gave final approval of the version to be published. GD: genetic analysis. All authors read and approved the final manuscript.

## Pre-publication history

The pre-publication history for this paper can be accessed here:

http://www.biomedcentral.com/1471-2431/14/188/prepub
